# Supplementary material for: Changes in the spatial and temporal pattern of natural forest cover on Hainan Island from the 1950s to the 2010s: implications for natural forest conservation and management
Source: PeerJ. 2017 May 16;5:e3320. doi: 10.7717/peerj.3320 (PMC5436588; doi:10.7717/peerj.3320)
Supplement: Appendices — Appendix 1 Description of obtaining the NFC in 1975 from 93 topographical maps Appendix 2 Description of the field investigation for estimating the accuracy of natural forest distribution Appendix 3 The sources of historical road network maps and road levels Appendix 4 Description of the measure of four road-related indicators Appendix 5 Comparing the predictive performances of four model techniques Appendix 6 Overlaying the distribution of NFC with the road network in four pattern predictive performances of four model techniques [file peerj-05-3320-s001.docx]

Supplementary Materials for

Changes in the spatial and temporal pattern of natural forest cover on Hainan Island from the 1950s to the 2010s：implications for natural forest conservation and management

This file includes:

**Appendix 1** Description of obtaining the NFC in 1975 from 93 topographical maps

**Appendix 2** Description of the field investigation for estimating the accuracy of natural forest distribution

**Appendix 3** The sources of historical road network maps and road levels

**Appendix 4** Description of the measure of four road-related indicators

**Appendix 5** Comparing the predictive performances of four model techniques

**Appendix 6** Overlaying the distribution of NFC with the road network in four pattern predictive performances of four model techniques

**Appendix 1 Description of obtaining the NFC in 1975 from 93 topographical maps**

We used 93 topographical maps that are incorporated different types of land uses and outlined the distribution of arboreal forest, shrub land, and open woodland to obtain the NFC in 1975 of Hainan Island.

We divide this process into three steps. First, we scan the 93 papery maps into digital maps, which are covering the whole Hainan Island. Coordinates of the four vertices of each map adopted as frame of references to define its existence in physical space by using the “*Georeferencing*” tool in ArcInfo 9.3. (ESRI 2008). Second, we drew polygons basic on the distribution borderlines of arboreal forest, shrub lands and open woodland shows in each map (Table S1). All those polygons were merged together became an whole polygon representing the distribution of natural forest in 1975. Third, the natural forest polygon was overlaid with the 5km×5km fishnet to calculate the natural forest cover (NFC) of each grid.

**Table S1** The symbols representing the arboreal forest, shrub land and open woodland in each map and obtained method

| Symbol | Sample in map | Referent | Obtained method |
| --- | --- | --- | --- |
| Polygons are marked in Green | 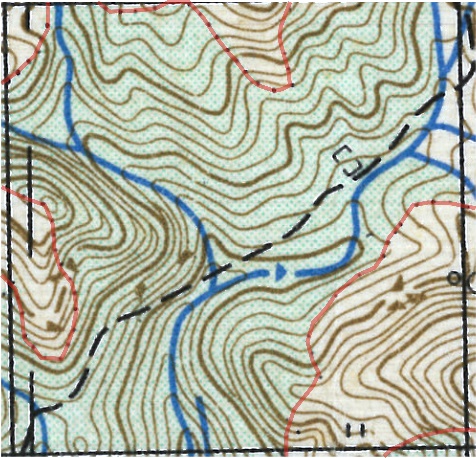 | Arboreal forest | We drew the borderline of the polygons are marked in green in each map. For example, the transparent line in red of the sample figure. Then all polygons representing arboreal forest have merged. |
| Polygons are around by dashed lined and marked by 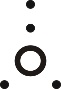 symbol. | 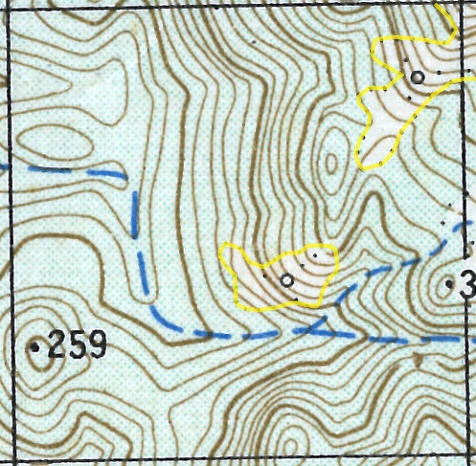 | Shrub land | We drew the borderline of the polygons are marked by 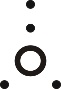 symbol in each map. For example, the transparent line in yellow of the sample figure. Then all polygons representing shrub land have merged. |
| Polygons are around by dashed lined and marked by 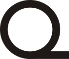 symbol. | 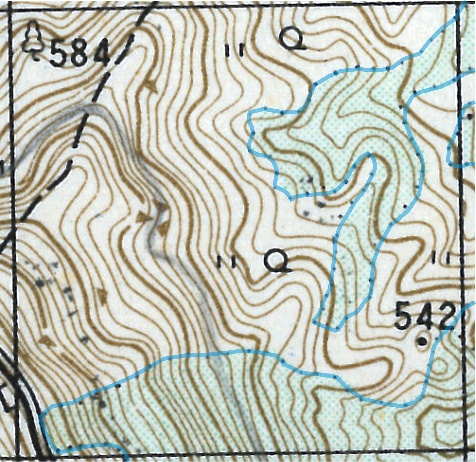 | Open woodland | We drew the borderline of the polygons are marked by 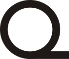 symbol in each map. For example, the transparent line in blue of the sample figure. Then all polygons representing open woodland have merged. |

**Appendix 2 Description of the field investigation for estimating the accuracy of natural forest distribution, and revising the misclassification.**

We conducted fieldwork campaigns to study wildlife in Hainan during 1997-1998 and 2012-2013 respectively (Xu et al. 2016). A stratified sampling method used to design the sampling points (Fig. S1), and the land use for each point was recorded in field. The accuracy measures of natural forest distribution extent in 1995 and 2012 derived and assessed by separately comparing that field investigation data with map data in a confusion matrix (Table S2; Table S3). Map data extracted for these points from the natural forest distribution map in 1995 and 2012. Field investigation data derived by field investigation who labelled each sample point with natural forest or other class.

The information of sample points not only to estimate the accuracy of our natural forest distribution extent in 1995 and 2012. When we found some misclassify. When we found the misclassification of those sample points between the map data and field investigation data. First, we gain the geographical coordinates of the misclassified sample point. Then, we used the satellites image within the investigation period to reshape the extent around the misclassified sample point. Additionally, the information of elevation, slope and aspect recorded in the field investigation also used as reference when revising the extent.

**
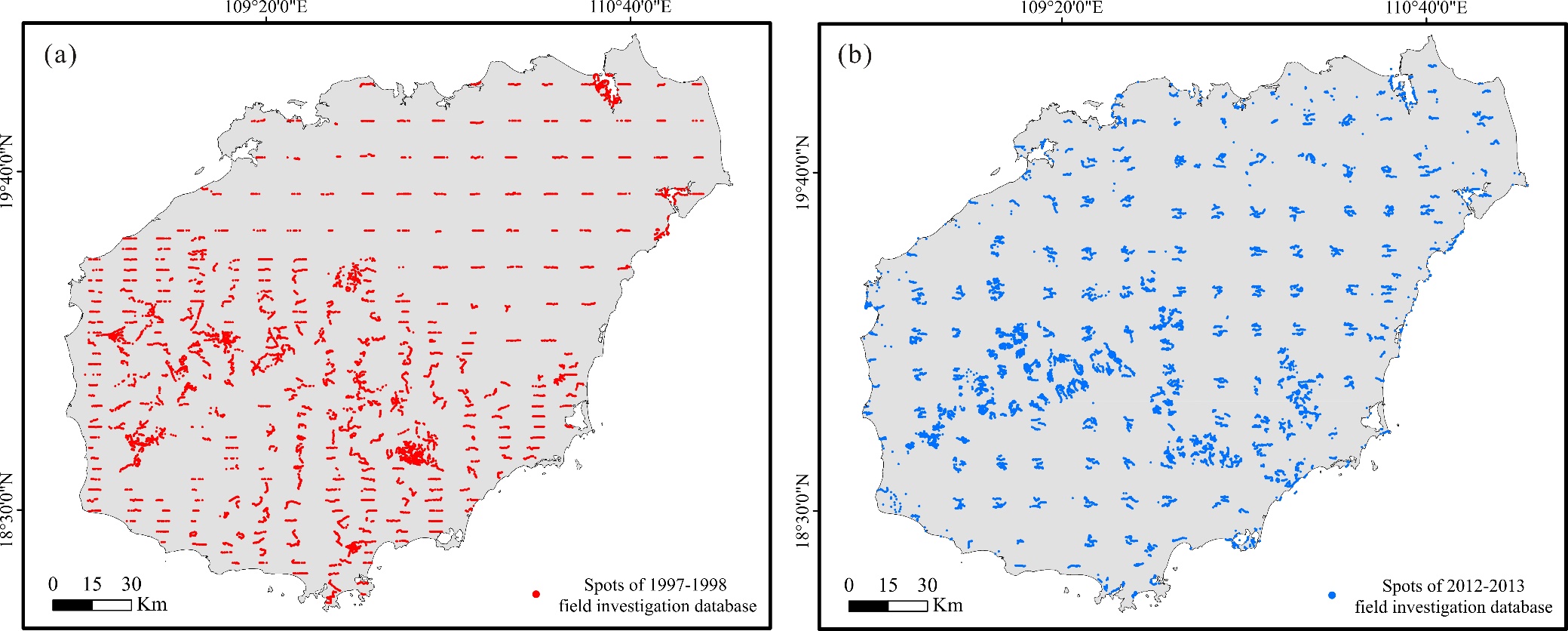
**

**Figure S1** The distribution of field investigation spots in (a) 1997-1998 and (b) 2012-2013

**Table S2** Confusion matrix for assessing the accuracy of natural forest distribution map in 1995. The matrix shows the field investigation (1997-1998) and map classification (1995) cases in the columns and rows, respectively. The proportion of correctly allocated cases indicates the overall classification accuracy.

| Classification of natural forest  distribution map in 1995 | Field investigation in 1997-1998 | | ∑map case | Accuracy (%) |
| --- | --- | --- | --- | --- |
|  | Natural forest | Others |  |  |
| Natural forest | 5574 | 1344 | 6918 | 80.6 |
| Others | 2821 | 7176 | 9997 | 71.8 |
| ∑Field investigation case | 8395 | 8520 |  |  |
| Accuracy (%) | 66.4 | 84.2 |  |  |
| Overall Accuracy (%) | | | | 76.2 |

**Table S3** Confusion matrix for assessing the accuracy of natural forest distribution map in 2012. The matrix shows the field investigation (2012-2013) and map classification (2012) cases in the columns and rows, respectively. The proportion of correctly allocated cases indicates the overall classification accuracy.

| Classification of natural forest  distribution map in 2012 | Field investigation in 2012-2013 | | ∑map case | Accuracy (%) |
| --- | --- | --- | --- | --- |
|  | Natural forest | Others |  |  |
| Natural forest | 9995 | 1504 | 11499 | 86.9 |
| Others | 2501 | 9668 | 12169 | 79.4 |
| ∑Field investigation case | 12496 | 11172 | 23668 |  |
| Accuracy (%) | 80 | 86.5 |  |  |
| Overall Accuracy (%) | | | | 83.2 |

**Appendix 3 The sources of historical road network maps and road levels**

The historical road network database in the 1950s, 1970s, 1990s and 2010s obtained from various map sources (Table S4). Different levels of roads that is higher than rural road are showed in those maps, including Expressway, Highway, simply-built highway, Cart road and Rural road (Table S4). Considering the function and surface material of those various roads, we classified those roads into two classes in the 1950s, 1970s, 1990s and 2010s severally for further analysis: main road (including Expressway, Highway and simply-built highway) and secondary road (including Cart road and Rural road).

**Table S4** The sources of historical road network maps

| **Represented**  **decade** | **Name of map** | **Type of map** | **Year of**  **map-making** | **Scale** | **Source** | **Included levels of road** |
| --- | --- | --- | --- | --- | --- | --- |
| 1950s | CHINA Topographic Maps  (Hainan Island part) | Papery | 1958-1960 | 1:250000 | Compiled by The Army Service(LU),  Corps of Engineers, U.S. Army | First class road (= Highway)  Second class road (= Simply-built highway)  Third class road (= Cart and Rural road) |
| 1970s | Topographical maps of Hainan  (in Chinese) | Papery | 1975 | 1:50000 | Compiled by Chinese People's Liberation Army | Highway, Simply-built highway,  Cart road and Rural road |
| 1990s | Road network of China  (Hainan Island part, in Chinese) | Digital | 1995 | 1:50000 | National Geomatics Center of China, available from http://ngcc.sbsm.gov.cn. | Expressway, Highway, Simply-built highway, Cart road and Rural road |
| 2010s | Trunk road network of Hainan  (in Chinese) | Digital | 2013 | 1:350000 | Compiled by Traffic Way Administration of Hainan Province, available from  http://www.hainangl.cn/ | Expressway, Highway, Simply-built highway, Cart road and Rural road |

**Appendix 4 Description of the measure of four road-related indicators**

We used four road-related indicators to represent the situation of road expansion and configuration, which is SRL, DNR, NON and MND (Table S5). Basic on the road network database, we calculated four road-related indicators following the measurements show in Table S5. In each decade, the road-related indicators of main and secondary road calculated singly.

**Table S5** Measure of four road-related indicators

| **Indicator** | **Formula** | **Units** | **Sketch map** |
| --- | --- | --- | --- |
| Sum of road length | SRL = $\sum_{1}^{i} d_{i}$ | *Meter* | 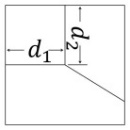 |
| Distance from the grid cell centroid to the nearest road | DNR = $a_{i}$ | *Meter* | 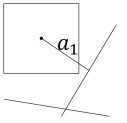 |
| Number of nodes of road network | NON = n | *Ea.* | 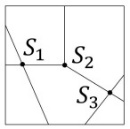 |
| Mean node degree (the mean number of roads connected to each node) | MND = $\frac{1}{N}\sum_{1}^{i} k_{i}$ | *Ea.* | 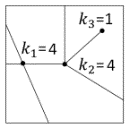 |

**Appendix 5 Comparing the predictive performances of four model techniques**

In order to find a feasible model technique in this case study, we compared the predictive performances of our model techniques in simulating the NFC of each gird in the 1970s, 1990s and 2010s, including generalized linear model (GLM), generalized additive model (GAM), artificial neural networks (ANN) model, and random forests (RF) model. We used the normalised mean square error (NMSE) to compare the predictive performance of the four models in simulating the NFC of each 5×5 km grid cell. NMSE is a relative measure, estimating the overall deviation between predicted and measured values and also a unit-less measure in the interval [0,1]. The modelling technique with the lowest value of NMSE has the highest predictive performance.

First, we used the NFC data of each grid cell in the 1970s, 1990s and 2010s which was measured from the maps data as dependence variable, ELE, SLO, CPP, and main and secondary road networks related indicators in the 1970s, 1990s and 2010s (SRL, DNR, NON and MND) as independence variables to train the model. Second, we used the training model with the same independence variables to predict the NFC data in the 1970s, 1990s and 2010s. At last, basic on the measured and predicted NFC in the 1970s, 1990s and 2010s, we calculated the NMSE value of each model (Table S6). The RF model showed the lowest NMSE among the four models (Table S6) and consequently, was used for reconstructing the historical NFC from the 1950s to the 2010s

**Table S6** The NMSE (Normalised Mean Square Error) value of four model techniques in predicting the NFC of each grid cell in the 1970s, 1990s and 2010s.

|  | **The 1970s** | **The 1990s** | **The 2010s** |
| --- | --- | --- | --- |
| GLM | 0.42 | 0.38 | 0.26 |
| GAM | 0.40 | 0.34 | 0.24 |
| ANA | 0.61 | 0.61 | 0.51 |
| RF | 0.16 | 0.14 | 0.06 |

**Appendix 6 Overlaying the distribution of NFC with the road network in four pattern predictive performances of four model techniques**

We overlay the distribution of NFC with the road network in each decade (Fig. S2) to explore how the road network determined the natural forest fragmentation patterns. The gradient colour from blue to yellow represent the NFC descending. The red line represents the main road network, whereas the black line represents the secondary road.


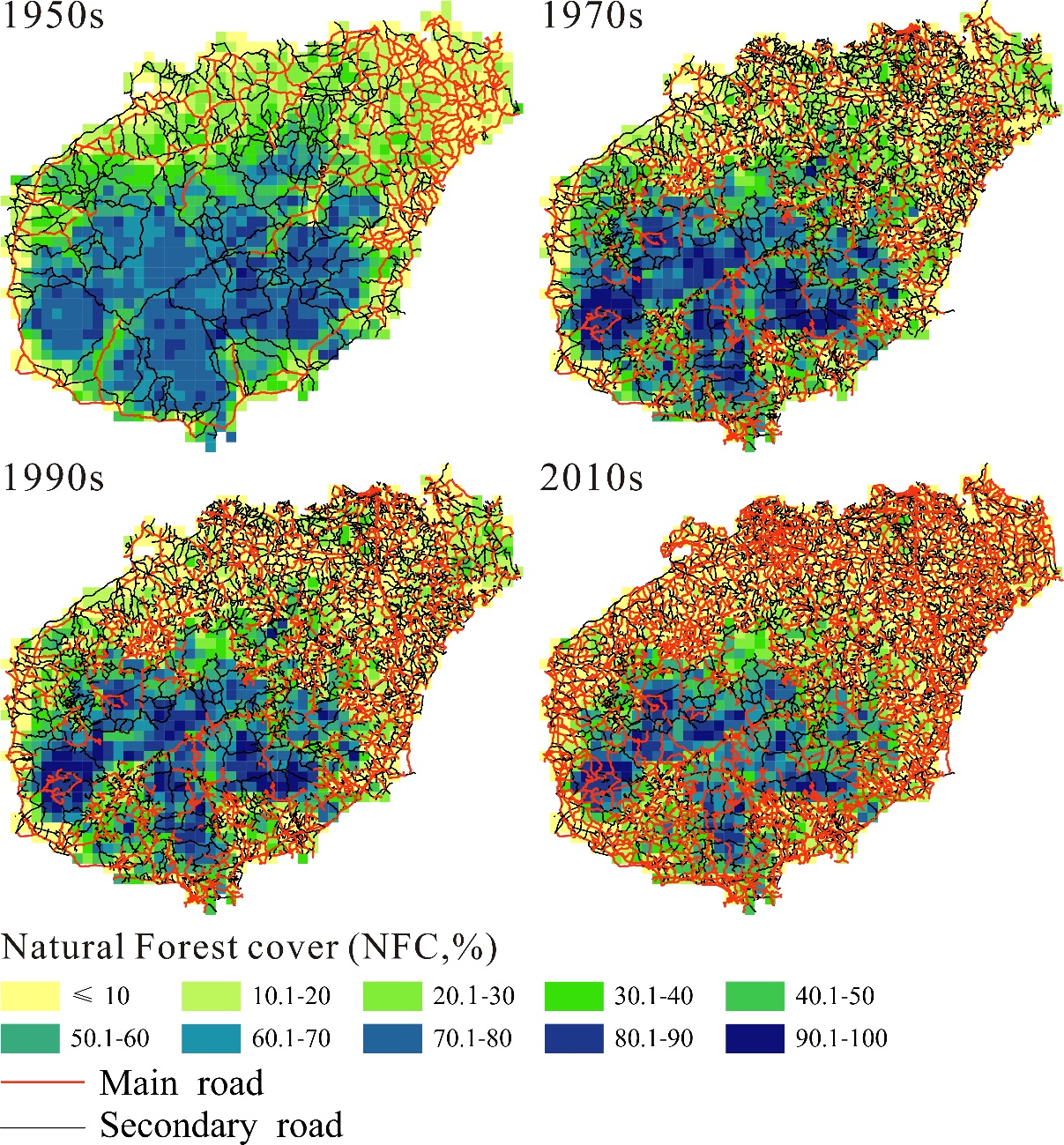


**Figure S2** Overlaying the road network with the distribution of NFC in each decades
